# A coordinated approach for managing polypharmacy among children with medical complexity: rationale and design of the Pediatric Medication Therapy Management (pMTM) randomized controlled trial

**DOI:** 10.1186/s12913-023-09439-y

**Published:** 2023-04-29

**Authors:** Lucas E. Orth, Chris Feudtner, Allison Kempe, Megan A. Morris, Kathryn L. Colborn, R. Mark Gritz, Sunny A. Linnebur, Anowara Begum, James A. Feinstein

**Affiliations:** 1 Skaggs School of Pharmacy & Pharmaceutical Sciences, University of Colorado Anschutz Medical Campus, Aurora, CO USA; 2 Department of Pharmacy, Children’s Hospital Colorado, Aurora, CO USA; 3 Division of General Pediatrics, Department of Pediatrics, Children’s Hospital of Philadelphia, Philadelphia, PA USA; 4 Departments of Pediatrics and Medical Ethics and Health Policy, University of Pennsylvania Perelman School of Medicine, Philadelphia, PA USA; 5 Adult & Child Center for Outcomes Research & Delivery Science (ACCORDS), University of Colorado Anschutz Medical Campus and Children’s Hospital Colorado, 1890 N. Revere Court, 3Rd Level, Mail Stop F443, Aurora, CO 80045 USA; 6 Department of Pediatrics, University of Colorado Anschutz Medical Campus, Aurora, CO USA; 7 Department of Medicine, University of Colorado Anschutz Medical Campus, Aurora, CO USA; 8 Department of Surgery, University of Colorado Anschutz Medical Campus, Aurora, CO USA; 9 Department of Biostatistics and Informatics, Colorado School of Public Health, Aurora, CO USA

**Keywords:** Pediatrics, Children with medical complexity, Polypharmacy, Deprescribing, Medication safety, Adverse drug events

## Abstract

**Background:**

Children with medical complexity (CMC) often rely upon the use of multiple medications to sustain quality of life and control substantial symptom burden. Pediatric polypharmacy (≥ 5 concurrent medications) is prevalent and increases the risk of medication-related problems (MRPs). Although MRPs are associated with pediatric morbidity and healthcare utilization, polypharmacy is infrequently assessed during routine clinical care for CMC. The aim of this randomized controlled trial is to determine if a structured pharmacist-led Pediatric Medication Therapy Management (pMTM) intervention reduces MRP counts, as well as the secondary outcomes of symptom burden and acute healthcare utilization.

**Methods:**

This is a hybrid type 2 randomized controlled trial assessing the effectiveness of pMTM compared to usual care in a large, patient-centered medical home for CMC. Eligible patients include all children ages 2–18 years old, with ≥ 1 complex chronic condition, and with ≥ 5 active medications, as well as their English-speaking primary caregivers. Child participants and their primary parental caregivers will be randomized to pMTM or usual care before a non-acute primary care visit and followed for 90 days. Using generalized linear models, the overall effectiveness of the intervention will be evaluated using total MRP counts at 90 days following pMTM intervention or usual care visit. Following attrition, a total of 296 CMC will contribute measurements at 90 days, which provides > 90% power to detect a clinically significant 1.0 reduction in total MRPs with an alpha level of 0.05. Secondary outcomes include Parent-Reported Outcomes of Symptoms (PRO-Sx) symptom burden scores and acute healthcare visit counts. Program replication costs will be assessed using time-driven activity-based scoring.

**Discussion:**

This pMTM trial aims to test hypotheses that a patient-centered medication optimization intervention delivered by pediatric pharmacists will result in lower MRP counts, stable or improved symptom burdens, and fewer cumulative acute healthcare encounters at 90 days following pMTM compared to usual care. The results of this trial will be used to quantify medication-related outcomes, safety, and value for a high-utilization group of CMC, and outcomes may elucidate the role of integrated pharmacist services as a key component of outpatient complex care programs for this priority pediatric population.

**Trial Registration:**

This trial was prospectively registered at clinicaltrials.gov (NCT05761847) on Feb 25, 2023.

**Supplementary Information:**

The online version contains supplementary material available at 10.1186/s12913-023-09439-y.

## Background

### Pediatric polypharmacy and children with medical complexity

Pediatric polypharmacy(defined as concurrent use of ≥ 5 medications) is a major public health problem with high prevalence among the priority population of children with medical complexity (CMC) [1]. Characterized by the presence of complex chronic conditions (e.g., intractable epilepsy, degenerative neurologic disease) that are expected to last at least 12 months and require subspecialty care or tertiary care hospitalizations, CMC often require treatment with complex polypharmacy to sustain quality of life and control substantial symptom burden [1-11]. Pediatric polypharmacy is shown to increase the risk of medication-related problems (MRPs) [11-28]. A MRP is an event involving medication therapy that interferes with an optimum patient outcome, for example, an inappropriate therapy, undertreated symptom, major drug-drug interaction, or adverse drug event (ADE) [12, 13, 16-28]. These types of MRPs are defined, measurable, and potentially treatable if recognized [12, 13, 16-29]. Although MRPs are associated with patient morbidity and healthcare utilization, polypharmacy is infrequently assessed during routine clinical care for CMC, and MRPs are managed ad hoc [10, 30-33].

While polypharmacy is often necessary for symptom and disease management in CMC, opportunities for improved outpatient medication management are ubiquitous [10, 30-33]. Current pediatric polypharmacy management strategies are fragmented and reactive, rather than proactive [34]. CMC are often prescribed medications by multiple sub-specialists and lack a coordinating medication supervisor [8]. Isolated medication regimen reviews may occur when CMC experience acute healthcare changes or ADEs [33]. In contrast, the Centers for Medicare & Medicaid Services requires Medicare sponsors to provide preventive medication therapy management (MTM) programs to targeted adult patients [35]. Standardized pharmacist-led MTM activities (e.g., medication optimization, deprescribing, education) are patient-centric, comprehensive, and improve health outcomes and safety [35-41].

Numerous potential benefits of a systematic approach to MTM-like services in an analogous pediatric population have been described. In a study of 100 CMC with polypharmacy in the ambulatory setting, an average of 3.4 MRPs were identified per patient, with 97% of patients having opportunities for potential intervention [12]. Most frequently proposed interventions included drug discontinuation trials, caregiver education, dose modification, and modification of dosage form or frequency to reduce medication regimen complexity. In a separate, health system-wide initiative focused on medication list reviews within a broad pediatric population, a group of ambulatory clinical pharmacists performed 409 interventions over a 6-month pilot period, most frequently involving the management of asthma, infections, or pain [42]. The majority of interventions resulted in full resolution of identified MRPs, but the authors described a need for further investigation to determine the value-based sustainability of the program.

In the priority population of CMC, the additional administrative complexity of polypharmacy regimens may introduce further risks and opportunities for benefit of MTM services, particularly those focused on medication simplification where appropriate. In a study of 123 pediatric patients with neurological impairment and polypharmacy, patients’ medication regimens included a median of 31 total doses of medication, 6 unique dosage forms, 7 different dosing frequencies, and 5 medications with additional administration specifications (e.g., split/crush tablet, open capsule for administration via g-tube) per patient [4]. Safety and effectiveness of these regimens is therefore highly dependent on caregiver understanding and ability. In a study of 156 caregivers of CMC, most parents were highly involved in home medication administration, but some reported concerns about medication administration and safety [9]. Of all caregivers, only 73% were able to correctly match a medication to its targeted symptoms, 60% were able to report complete dosing instructions, and 55% were able to correctly measure liquid medication doses. Significant differences existed between caregivers’ perceived understanding of such abilities versus demonstrated task performance. Related concerns have been described by parents and investigators elsewhere [15, 34, 43].

### Major knowledge gaps and research needs

In 2021, the Joint Commission Sentinel Event Alert highlighted the dire need for “additional research on interventions to reduce pediatric medication errors, especially in emergency departments, ambulatory clinics and home environments” [44]. Despite a robust body of prior research demonstrating the risks of pediatric polypharmacy, rigorously tested pediatric-specific interventions to manage polypharmacy-related issues are scarce and greatly needed [10, 30-33]. Complex care programs that provide comprehensive care to CMC have identified pharmacy support as a preeminent need [33]. While medication safety is a priority for pediatric complex care programs, a systematic intervention will not be widely adopted without demonstrated effectiveness and value for CMC [45]. Pharmacists may provide targeted reactive pharmaceutical care in the existing model, but proactive comprehensive care is needed [42, 46-49]. Pediatric pharmacy specialists currently provide support in multiple hospital settings, but pediatric pharmacists are infrequently incorporated into outpatient models of care for CMC [32, 42, 46, 47, 50]. However, a more central role has been proposed for outpatient pediatric pharmacists in the medical home to coordinate and manage medication regimens, and to support primary care providers (PCPs) [33, 48, 51]. Furthermore, parental acceptance of this model is high; in the previous study of 156 parents of CMC with polypharmacy, 87% were willing to change ≥ 1 medication(s) if recommended by their provider [9].

As care models evolve, thoughtful incorporation of proactive and preventative evidence-based strategies into the management of pediatric polypharmacy is necessary to improve medication-related patient outcomes, safety, and value. Pharmacist-led MTM is a proven and effective tool for managing adult and geriatric polypharmacy [35-41]. The overarching aim of this trial is to determine if a structured pharmacist-led Pediatric Medication Therapy Management (pMTM) intervention will improve the proactive management of polypharmacy in CMC by directly addressing major gaps in current practice.

### An approach for improving the management of pediatric polypharmacy

We propose a rigorous and efficient hybrid type 2 trial with evaluation of pMTM guided by the RE-AIM (Reach, Effectiveness, Adoption, Implementation, and Maintenance) framework [52] with the following specific aims:

- *Aim 1*: Assess Reach and Effectiveness by determining the effect of a pMTM intervention on the primary outcome of total MRPs among CMC with polypharmacy, as well as the secondary outcomes of parent-reported symptoms and acute healthcare utilization, compared to usual care. We hypothesize that pMTM will result in lower MRP counts, stable or improved symptom burdens, and fewer cumulative acute healthcare encounters compared to usual care.
- *Aim 2*: Determine how key patient and parent factors modify pMTM Effectiveness through quantitative measurement of the effect modification of patient/parent factors on the primary MRP outcome, as well as through qualitative parental report. We hypothesize that higher medical complexity and higher parental health literacy will be associated with a larger treatment effect.
- *Aim 3*: Evaluate provider pMTM Adoption, Implementation, and potential for Maintenance through assessment of actual provider adoption, fidelity/time requirements, qualitative provider perceptions (including feasibility, acceptability, and barriers or facilitators), and assessment of program replication costs.

Through a systematic approach, the results of this pMTM trial will inform the medical community on the value and effectiveness of pMTM towards optimization of polypharmacy among the priority population of CMC.

## Methods/D﻿esign

### Protocol reporting

This protocol has been prepared according to the RE-AIM framework (Table 1) and the Standard Protocol Items: Recommendations for Interventional Trials (SPIRIT) Statement (Table 2) [52-54]. Trial results will be reported according to the Consolidated Standards of Reporting Trials (CONSORT) and the Consolidated Criteria for Reporting Qualitative Research (COREQ) guidelines [55-57]. This trial was registered at clinicaltrials.gov (NCT05761847) on 02/25/2023. The SPIRIT Checklist is provided as Additional File 1.

**Table 1:** Outcome measures and measurement strategies using the RE-AIM framework

		Outcome Measure	How We Will Measure (Data Source)
**Aim**	**Reach**	1. CMC with polypharmacy who participate in pMTM	1. % and representativeness of patients who participate vs. decline (EHR)
**1**	**Effectiveness**	1. **Medication-Related Problems (MRPs)** 2. Parent-Reported Outcomes of Symptoms (PRO-Sx) 3. Acute healthcare utilization	1. **MRP count at 90 days (EHR)** 2. PRO-Sx total symptom score (parent) 3. Acute visit count at 90 days (EHR)
**2**	**Effect Modification**	MRP count associated with: 1. Patient medical complexity 2. Parent health literacy 3. Barriers/facilitators of parental implementation	1. ICD-10-CM codes (EHR) 2. Short Assessment of Health Literacy (parent) 3. Qualitative interviews (parent)
**3**	**Adoption**	1. Providers’ participation in pMTM	1. % and representativeness of those who participate vs. decline (annual survey)
	**Implementation**	1. Fidelity of key pMTM components 2. Time required by providers to implement pMTM 3. Providers’ perceived barriers/facilitators of pMTM implementation	1. Audio-recorded encounters (audio) 2. Time spent on medication-related activities during clinical visit (audio) 3. Qualitative interviews (provider)
	**Maintenance**	1. Healthcare teams’ perceptions of and intentions regarding continuing pMTM following the trial 2. Overall pMTM replication program costs	1. Interviews with providers and leadership 2. Time-driven activity-based costing related to implementation and maintenance of pMTM relative to usual care costs

**Table 2:**
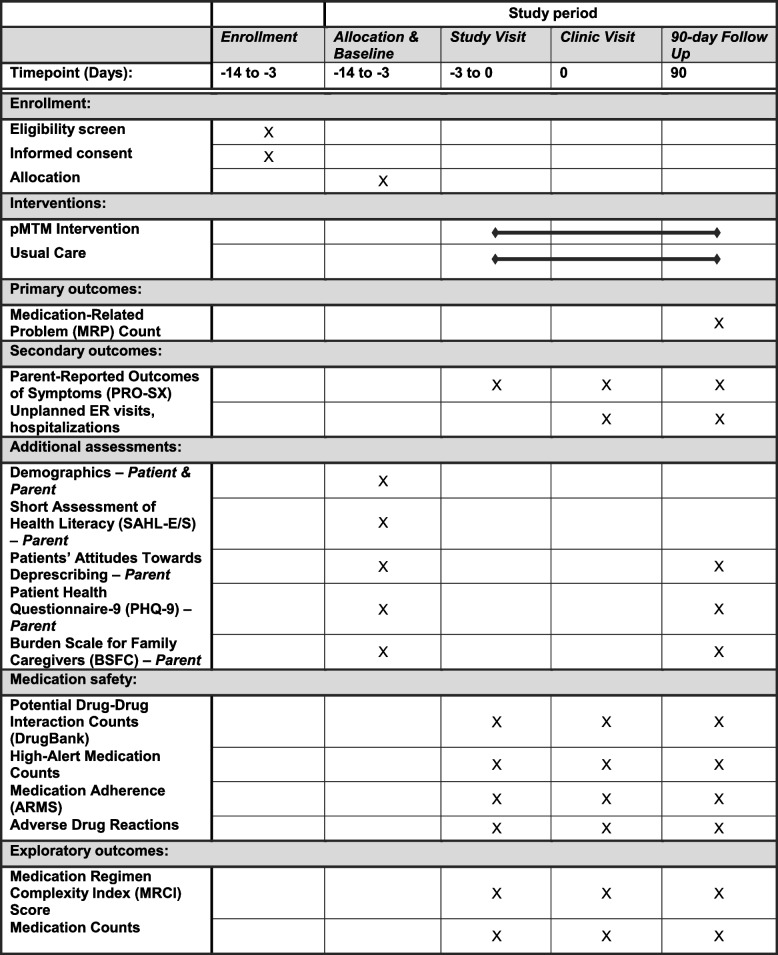
Enrollment, interventions, and assessments according to the Standard Protocol Items: Recommendations for Intervention Trials (SPIRIT) diagram

### Trial design

This trial is a 5-year hybrid type 2 randomized controlled trial funded by the Agency for Healthcare Research and Quality (AHRQ) and designed to evaluate the management of pediatric polypharmacy in the primary care setting by comparing the pMTM intervention to usual care for lowering the primary outcome of MRP counts and secondary outcomes of symptom burdens and acute healthcare utilization. Because pediatric pharmacist support is currently a limited resource, a hybrid type 2 design is the most efficient, rigorous design to simultaneously evaluate the effectiveness and implementation of pMTM to enable rapid dissemination [58]. The intervention is not blinded to enrolled patients; however, study team members involved in assessment of outcomes, data analysis, and safety monitoring will be blinded.

All study procedures were reviewed and approved by the affiliated Institutional Review Board (IRB). Protocol amendments will be approved by the local IRB. Other pertinent parties will be notified through updates to the clinicaltrials.gov website. All publications related to the study will include a summary of protocol amendments.

### Study setting, participants, and eligibility criteria

Study enrollment is scheduled to begin in August 2023 and will occur through September 2027. The study will take place at the Special Care Clinic (SCC) at Children’s Hospital Colorado, a large multidisciplinary primary care medical home for CMC within a large, tertiary, freestanding children’s hospital. Patients ages 2–18 years old with ≥ 1 complex chronic condition and ≥ 5 concurrent medications (including prescription, as needed, and over-the-counter medications), and their primary parental caregiver will be screened for inclusion [5]. Patients with a non-English speaking primary caregiver will be excluded, as the pMTM intervention and certain study instruments are currently available only in English. Females and males and members of all racial and ethnic categories will be included if eligible, without bias.

### Randomization, allocation, and study phases

Eligibility screening will be conducted by trained research personnel using automated daily electronic health record (EHR) reporting tools that identify eligible children with a scheduled routine clinical visit in the SCC within the next 14 days. Following review of eligibility criteria, research personnel will contact the caregiver to introduce the study, invite the caregiver (and assenting adolescents) to participate, and obtain written consent.

Following consent, research personnel will work with study participants to complete baseline and 90-day assessments using EHR functionality. Baseline assessment will include patient and parent demographics, assessment of health literacy, assessment of parent attitudes towards deprescribing, and parental assessment of symptom burden (Table 2). Using the current EHR medication list, all participants will undergo medication history review with a study team member trained using WHO’s Standard Operation Protocol [59]; data will be collected for research purposes only, but if significant medication safety concerns are noted, the study team will alert the primary care provider (PCP) before the clinical visit. All additional data will be conducted during subsequent study and clinical visits.

**Fig. 1:**
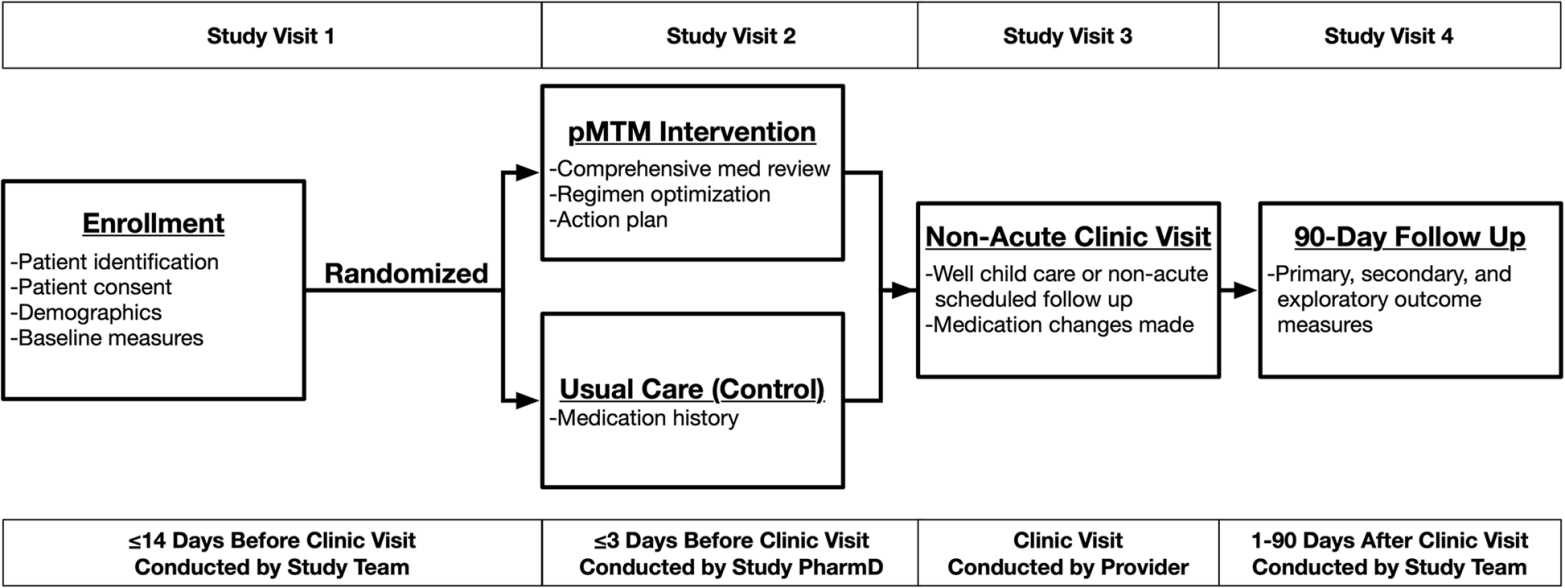
Study Flow Diagram. Protocolized study flow is depicted. Following enrollment and consent, participants will be randomized 1:1 to pMTM intervention or usual care, with the pMTM intervention occurring ≤ 3 days before a scheduled well child visit or routine follow-up medical encounter. Both groups will then be seen for their scheduled PCP visit as occurring within usual care. Applicable outcomes will be re-assessed at 90-day follow-up

Participants will then be randomized 1:1 in permuted blocks of 4 patients to pMTM intervention or usual care (2 patients to each arm), with the pMTM intervention occurring ≤ 3 days before a scheduled well child visit or routine follow-up medical encounter (Fig. 1). Those randomized to intervention will meet with a study pharmacist (PharmD) in-person or via telehealth for completion of the pMTM encounter (described comprehensively below). Both groups will then be seen for their scheduled PCP visit as occurring within usual care. After the clinical visit, all participants will receive the post-clinical visit medication list and, for those in the intervention arm, the medication action plan (MAP). Participants will be followed for 90 days after the clinical visit to track the primary, secondary, and exploratory outcomes (Tables 2 and 3).

**Table 3:** Assessments and outcomes supporting pMTM effectiveness aims

Minimum Data Elements for Aims 1 & 2
**Baseline Assessments:**
• Demographics – *Patient & Parent*
• Complex Chronic Conditions (ICD-10) [5] – *EHR*
• Medication history – *EHR, Parent*
• Short Assessment of Health Literacy (SAHL-E) [63] – *Parent*
**Primary Outcome at 90 Days:**
• Medication-related problem count [12] – *EHR, Parent*
**Secondary Outcomes at 90 Days:**
• Parent-Reported Outcomes of Symptoms (PRO-Sx) [7]
• Acute healthcare utilization visit count [1, 13] – *EHR*
**Exploratory Outcomes at 90 Days:**
• Medication Regimen Complexity Index Scores [4]—*EHR*
• Medication counts [1]—*EHR*

### Treatments

#### Intervention: pMTM conceptual framework

**Fig. 2:**
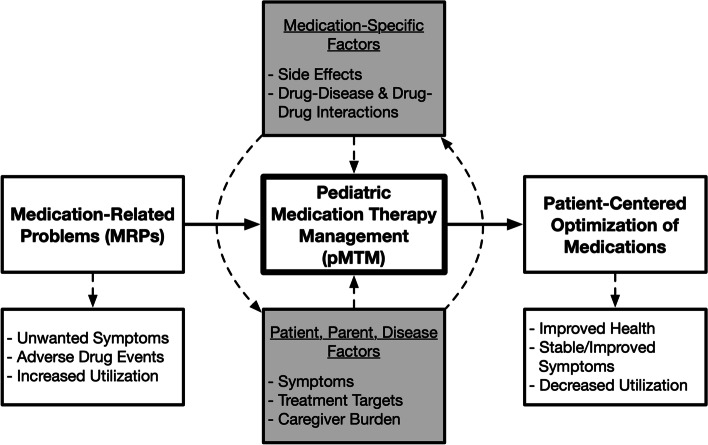
Conceptual Framework for pMTM Intervention. This protocol is conceptualized based on the SHED-MEDS model of deprescribing [60]. With permission, we modified this model with the broader core activity of pMTM, during which parents and providers review medication changes, continuation, proper use, monitoring, and follow-up. For children with polypharmacy, our model specifies that the pMTM intervention (which accounts for and prioritizes safety, quality of life, and parental considerations) will lead to patient-centered optimization of medications, resulting in improved health and symptoms manifest as fewer MRPs [4, 12, 30].

The trial design is conceptualized based on the Shed-MEDS model of deprescribing, which posits that adult patients with potentially inappropriate polypharmacy will benefit from a patient-centered deprescribing intervention to reduce polypharmacy and improve health [60]. With permission, the model is modified to include the broader core activity of pMTM, during which parents and providers review medication changes, continuation, proper use, monitoring, and follow-up (Fig. 2) [30, 60]. For CMC with polypharmacy, our model specifies that the pMTM intervention (which accounts for and prioritizes safety, quality of life, and parental considerations) will lead to patient-centered optimization of medications [4, 30, 61].

#### Intervention: pediatric medication therapy management steps

Following baseline patient and caregiver assessments, patients randomized to the intervention will take part in a PharmD visit for application of the pMTM intervention, occurring in person or via telehealth within 3 days prior to the planned PCP clinical visit. The pMTM intervention will be applied during a 30- to 45-min visit in which the PharmD will work collaborative with the caregiver to complete 3 major activities (Table 4).

**Table 4:** Components of comprehensive pMTM Intervention

Major Activity	Specific Components	Duration
**Comprehensive review of medication regimen**	1. Update medication list	10–15 min
	2. Review patient/caregiver goals for each therapy	
	3. Confirm adherence or identify barriers to adherence (e.g., cost, taste aversion, difficult administration)	
	4. Review current effectiveness for any targeted symptoms or lab values	
	5. Identify therapeutic duplication and/or potentially unnecessary therapies	
	6. Review for potentially medication-attributable symptoms or adverse effects	
**Optimization of medication regimen**	1. With caregiver, identify list of ongoing concerns – could be ADEs, symptoms perceived to be undertreated, less-than-ideal administration schedule. *Prioritize this list according to (1) safety, (2) patient quality of life, (3) caregiver/family quality of life*	10–15 min
	2. Formulate plan for medication adjustment (e.g., dose optimization, schedule change, formulation change), discontinuation, or initiation to address above issues. *May be list of medication changes today or a detailed plan for recommendations to take back to a subspecialist*	
	3. Include expectations for both subjective monitoring and objective labs or tests (e.g., ECG) that will be necessary/recommended, with expected timeline	
**Creation of medication action plan**	1. Caregiver-friendly medication list (directly populated from EHR)	5–10 min
	2. Bulleted list of current changes (to make today) and planned changes	
	3. Plan for follow-up with pharmacist or another provider	

First, the PharmD will perform with the patient and caregiver a comprehensive medication review (CMR) of the patient’s current personal medication list (PML). CMR is a systematic process of collecting patient-specific data and assessing for potential medication-related problems. Subsequent clinical decisions are reliant on accuracy of available data; therefore, the first step of CMR is to conduct a thorough medication history using all available resources, updating the patient’s PML where necessary, and documenting that such activities have occurred. The PharmD will gather a list of active medications (including prescriptions, over-the-counter medications, dietary supplements, and complementary medicines), determine and confirm active disease states, and identify providers involved in the prescribing and management of current medication therapy. Next, the PharmD will review each disease therapy with the caregiver and patient, if appropriate, to determine current goals of therapy. Caregiver understanding of goals of therapy is important to their ability to provide confident care to CMC. Education related to mismatch of therapy goals and known medication effects may be addressed at this time. Additionally, the PharmD will determine if any barriers may be affecting adherence. Barriers to adherence in CMC often include taste aversion, difficult or confusing administration techniques, burdensome dosing schedule, or medication cost, among others. Such barriers may be addressed in subsequent steps of the pMTM intervention. Following review of therapy goals and adherence, the PharmD will evaluate any available laboratory data and discuss ongoing clinical symptoms to determine current medication effectiveness or lack thereof. The PML will be appraised for potential therapeutic duplication and therapies for which resolution of symptoms may render ongoing treatment unnecessary. Common examples of duplication of therapy within this population include use of multiple NSAID agents or acetaminophen-containing products or use of multiple medications within the same therapeutic class (e.g., clonidine and guanfacine). Such duplication may cause potentiation of adverse effects or contribute to excessive medication costs without conferring additional therapeutic benefit. As the final component of CMR, the PharmD will review current patient symptoms to identify those which may be attributable to medication use or toxicity, which may result in recommendations for alternative therapy or deprescribing. Gaps in therapy for guideline-directed care of various disease states (e.g., asthma) may also be identified at this stage.

The second essential element of the pMTM intervention involves optimization of the medication regimen to address those concerns or opportunities identified within the preceding CMR. With the caregiver, a list of all ongoing concerns will be prioritized according to goals of therapy. Considerations related to safety, patient quality of life, and caregiver or family quality of life will be measured during this process and communicated within subsequent recommendations for medication optimization. Next, the PharmD will formulate a plan for recommended medication-related changes, including potential dose or frequency adjustment, discontinuation of therapy, or initiation of alternate medications to manage untreated or ongoing disease symptoms. Recommendations will be classified according to type (Table 5), and rationale will be provided. All recommendations will be included in a structured pMTM provider note within the EHR which is intended to be informative and suggestive, as well as concise and respectful. Recommendations may be provided in the form of changes recommended for urgent action by the PCP (at the impending clinic visit) or communication with subspecialists for those diseases states primarily managed by alternate providers. Expectations and recommendations for both subjective monitoring and objective labs or tests (e.g., ECG) will be communicated to the caregiver and documented within the structured pMTM provider note. Any medications changes recommended during the visit will ultimately be made at the discretion of the PCP during clinical care.

**Table 5:** Types of interventions recommended within pMTM intervention

Modification (dose, form, frequency)
Discontinuation
Change to alternative therapy
Initiation of new therapy
Laboratory monitoring for safety/efficacy
Patient/caregiver education

As the final component of the pMTM intervention, after the clinical visit, the PharmD will create a written, patient-centered, and caregiver-friendly MAP using a template populated from the EHR. This MAP will describe a prioritized list of specific action items resulting from the interactive pMTM consultation, which empowers the caregiver to be personally involved in the administration of the proposed optimization(s). The document was developed from the CMS standardized format to allow for tracking of patient progress, clarification of intended patient response, and documentation of the perceived clinical effects of all changes [62]. The MAP is designed assist the caregiver with resolving current drug therapy concerns and to help achieve the goals of medication treatment but is not intended to provide the level of detailed communication provided to the PCP or other healthcare providers. Items reinforcing compliance, maintaining caregiver actions, and acknowledging success in the child’s medication therapy may be included. The caregiver will be encouraged to bring the MAP with them to future healthcare visits and to request update of the document as necessary. A plan for follow-up all changes with the PharmD of other appropriate providers will be outlined within the MAP and communicated to the caregiver. Additionally, the reconciled PML (created within the CMR stage of the pMTM visit) will be provided to the caregiver to assist in the understanding of current medication treatment and the tracking of potential medication changes, such as addition of over-the-counter medications or redaction of discontinued products. Information about appropriate disposal of unneeded medications will be provided by the PharmD if applicable.

#### Control group: usual care

Patients randomized to usual care will undergo medication history review performed by study personnel prior to the PCP clinical visits as previously described. The goal of this medication review process is to ensure accuracy of the baseline medication-related data without recommendations related to medication management. We selected a usual care comparator because there are no current established standards for centralized medication management strategies within the population of CMC. All medications decisions for the control group are at the discretion of the PCP.

### Study measures and data collection

Table 2 includes all study measures and data collection time points. To promote study retention, participants will receive compensation in the form of $50 gift cards at two study time-points (i.e., completion of clinical visit and at 90 days). Each study measure listed in Table 2 is briefly described below.

#### Demographics, health literacy, and attitudes towards medication management

Research personnel will use a standardized approach to extract information from the EHR related to basic patient demographics (age, gender, complex chronic conditions, level of polypharmacy). Complex chronic condition data is generated using the CCC V2 published classification system based on ICD-10 diagnosis codes [5]. Prior work has demonstrated that CMC with some complex chronic conditions, such as technology dependence (e.g., tracheostomy dependence, gastrostomy tube), may be exposed to higher levels of polypharmacy, and subsequent analysis will seek to determine if the medical complexity of CMC is associated with varying trial outcomes [1].

Caregiver health literacy will be assessed through parental completion of the Short Assessment of Health Literacy (SAHL-E) [63]. This test consists of 18 items, for which participants are instructed to read a medical term aloud before associating each term to another word with similar meaning to demonstrate comprehension. In this study, scores > 14 will indicate adequate health literacy, while scores ≤ 14 will indicate inadequate health literacy. Studies of medication management in adults have demonstrated a clear link between health literacy and medication self-management skills [64, 65]. Parents with different levels of health literacy may have different levels of engagement with the pMTM intervention, especially because medication optimization is comprised of multiple activities and not solely medication discontinuation. Ultimately, interventions to manage polypharmacy must support parents of all levels of health literacy [66-73]. While the pMTM intervention uses patient-centric communication modalities, understanding differences in the outcome by level of parental health literacy will guide post-trial refinements.

Finally, attitudes toward medication management will be assessed through parental completion of the Patients’ Attitudes Toward Deprescribing (PATD) tool [74]. This scoring system consists of 15 items used to classify the participant’s feelings towards polypharmacy, their own medication history, and comfort with discontinuation of medications.

#### Primary outcome measure: medication-related problems

The primary outcome is the MRP count at 90 days after the clinical visit during which the PCP finalizes any clinical recommendations for either the pMTM intervention or usual care groups. Because medication changes require time to effect change in clinical outcomes, we will collect outcome measurements at 90 days after the clinical visit, consistent with adult literature using the MRP outcome [12, 13, 16-28]. Robust evidence exists to support the utility of using MRPs as an outcome to evaluate MTM. As related to this outcome, we will follow established guidelines for analyzing and reporting composite measures [75-77]. To facilitate blinded assessment of MRPs, we will generate an EHR-based clinical summary at 90 days, including the current weight, active medication list, symptom report, lab values, serum levels, and any diagnostic codes related to ADEs (Table 6). Trained study personnel will contact parents to verify the medication history, symptom data, and any adverse events or acute healthcare utilization. Blinded outcome assessments will be made by ≥ 2 pediatric pharmacists not involved in the pMTM intervention using our published standardized approach [61].

**Table 6:** Common medication-related problems in children with medical complexity

Inappropriate or unnecessary therapy
Suboptimal therapy
Undertreated symptoms
Adverse drug event
Contraindicated drug-drug interaction
Duplication of therapy
Unclear prescription instructions

#### Secondary outcome measures: Parent-Reported Outcomes of Symptoms (PRO-Sx), and healthcare utilization

We will also measure changes in total parent-reported outcomes of symptoms (PRO-Sx) scores and 90-day acute healthcare utilization for both the intervention and usual care groups [6, 7, 78]. To ensure that symptoms are stable or improved after medication changes, we will track PRO-Sx scores, which we have experience measuring among CMC in the ambulatory setting [6, 7, 78]. Based on our prior work, it is feasible for parents to easily track symptoms from home via EHR functionality [6, 7, 78]. This will occur at scheduled time points, including the date of the pMTM visit for patients allocated to the intervention group, the date of the PCP clinical visit for all patients, and at 7 days, 30 days, and 90 days following the clinical visit. As part of the 90-day clinical summary, we will track counts of unplanned acute care utilization, including ambulatory sick visits, emergency room visits, and inpatient hospitalizations.

#### Exploratory outcomes and safety measures

During the 90-day follow up period, we will assess additional exploratory outcomes including Medication Regimen Complexity Index (MRCI) scores and medication counts for all medications, including scheduled, as needed, and over-the-counter medications [79]. MRCI, a tool developed to measure medication complexity in adult and geriatric populations with polypharmacy, has demonstrated potential for application in pediatric populations and has been associated with increased acute healthcare utilization [4, 61, 80-82]. In addition to the previously described patient-level PRO-Sx symptom burden scores, parental completion of the Patient-Health Burden Scale for Family Caregivers (BSFC) and Patient Health Questionnaire (PHQ-9) will be performed at scheduled time points to measure caregiver burden and mental health [83, 84].

In addition to assessment of ADEs as described within the MRP primary outcome, several other measures of medication safety and adherence will be collected. First, the DrugBank database will be interrogated against baseline and 90-day patient medication lists for potential drug-drug interaction count [85]. High-alert medication counts will be assessed using published guidance from the Institute for Safe Medication Practices [86]. Finally, medication adherence will be measured using the Adherence to Refills and Medications Scale (ARMS) at similar study time points [87].

#### Additional outcomes within RE-AIM framework

As defined within the RE-AIM framework of the study design, the aims of this study will address several goals which are not formally captured by the primary and secondary outcomes defined above, which primarily measure pMTM effectiveness. The outcomes that will be used to assess the impact of the pMTM intervention towards other aims are described briefly below:

##### Reach

Reach of the pMTM intervention will be quantified by measuring the percentage and representativeness of CMC with polypharmacy who accept and decline participation in the pMTM intervention. Study personnel will track the patients and parents declining participation, including previously defined demographics and reasons for non-participation.

##### Effect modification

Previously described variables of medical complexity, health literacy, and attitudes towards medication management will be assessed at the patient- and parent-level, where appropriate, to quantitatively evaluate intervention effect modification. To qualitatively evaluate effect modification, we will conduct a semi-structured interviews through the study period with a total of 40 caregivers. Qualitative interviews will include 10 caregivers from each subgroup (technology dependent/independent and high/low health literacy); only caregivers who participated in the pMTM intervention arm will be included. To recruit for this portion of the study, participating parents will receive a $25 gift card. A trained professional qualitative study team member will conduct recorded 1-h parent interviews via phone or video software. Qualitative interview guides will be pilot tested prior to use with study subjects. The guide will elicit parents’ perceptions of the feasibility, acceptability, and barriers/facilitators of the pMTM intervention, specifically focusing on how outcomes may have been impacted by their health literacy and whether their child was dependent on specific forms of technology. Recruitment for qualitative caregiver interviews will discontinue if ongoing analysis (described below) reveals thematic saturation [88].

##### Adoption, implementation, and maintenance

To measure PCP adoption of the pMTM intervention, short annual confidential surveys will be administered to quantify adoption of the pMTM intervention and recommendations by clinical providers, as well satisfaction and time spent related to pMTM. The study team will pilot test and monitor surveys to identify potential problems that could result in missing responses. Providers will be encouraged to complete all items on the survey, informed of the negative impact of missing data on the research, and assured that their answers are completely confidential. Those who participate will receive a $10 gift card after completing each annual survey. We will calculate the percentage and representativeness of eligible providers involved in the pMTM intervention and attempt to collect reasons for declination if observed.

Implementation fidelity will be evaluated through audio recording of a sample of visits from the intervention arm (pMTM visit and corresponding clinical visit) and the usual care arm (clinical visit). We will screen and recruit participants for recording of visits using permuted block randomization for a total of 100 audio-recorded encounters (50 encounters per arm). For in-person visits, study personnel will start the recorder and leave the room. The parent, child, or provider can stop recordings at any point. For telehealth-based pMTM study visits, audio recording will occur within the software. The audio-recorded clinical encounters will be used to compare whether the provider addresses pMTM-related components (medication review, optimizations, and action plan) during the clinical visits (binary outcome), and to estimate the time needed to implement the pMTM intervention or discuss medication-related issues (continuous outcome), focusing on differences between pMTM and usual care.

To measure aims related to pMTM maintenance, we will conduct 15 qualitative interviews with consented providers at time points including the beginning, middle, and end of the trial, for a total of 45 interviews. To reduce bias, we will attempt to interview all providers at least once during the study period. We will also attempt to interview some providers (specifically the pharmacists) at > 1 time point to evaluate how their experience with pMTM changed over time. Qualitative interview guides will be pilot tested prior to use with study subjects. We will elicit providers’ perceptions of the feasibility, acceptability, and barriers/facilitators of the pMTM intervention. At the final time point, we will specifically focus on providers’ perceptions and intentions of sustaining the interventions following the completion of the trial. Providers who participate in an interview will receive a $50 gift card. Recruitment will discontinue if ongoing analysis (described below) reveals thematic saturation [88].

Finally, to measure maintenance outcomes related to program replication costs, we will use time-driven activity-based costing approach to measure the cost related to implementation and maintenance of pMTM relative to usual care costs. Using best practices, we will develop process maps for patient/parent flow for both pMTM and usual care delivery and specify care activities and who (pharmacist, provider, other clinic staff) performs each activity [89]. The largest component of cost will be the time clinic staff devote to delivering pMTM and usual care, which we will measure using the audio recordings of clinical visits, annual surveys (questions about average time spent for pre-clinical visit preparation and post-visit documentation), and provider interviews (to explore reasons for variation) described above. Measures of time will be converted to cost using internal salaries and fringe benefits for each category of clinic staff. We will also value time using Bureau of Labor Statistics data to estimate more representative replication costs [90]. We will obtain cost information for other clinic and informatics resources directly and indirectly supporting the adoption, implementation, and maintenance of pMTM [91, 92].

### Blinding

Due to the nature of the pMTM intervention, patient, pharmacist, and PCP participants are not blinded to the intervention. Investigators and statisticians performing data analysis will be blinded to subject allocation. Additionally, participants involved in assessment of safety measures and pediatric pharmacists involved in assignment of the MRP primary outcome will be blinded to patient group assignment.

### Statistical methods

Patient and parent characteristics in both study arms will be evaluated using appropriate measures of central tendency and spread for continuous variables and proportions for categorical variables. For the primary MRP outcome analysis, we will assess for differences in MRP counts between the intervention and control groups at 90 days using generalized linear models with Poisson response distribution and log link function. The overall effectiveness of the intervention will be assessed by testing the model coefficient for randomization group, with a null hypothesis of no mean difference in MRP counts at 90 days between treatment and control groups. Model checking and diagnostics will be performed to assess validity of model assumptions, with appropriate remedial measures taken as necessary. For the secondary outcome analysis, we will assess for differences in outcome changes over time between the intervention and control groups using generalized linear mixed models, which accounts for correlation between repeated outcome measurements over time. Within-subjects correlation will be accounted for using a random intercept.

Towards assessment of patient and parent factors modifying pMTM effectiveness, we will employ similar generalized linear mixed models. Each of the pre-specified effect modifiers will be modeled as an interaction term between the intervention variable (binary) and the effect modifier variable (binary). The test of the null hypothesis that the interaction term’s coefficient is equal to 0 will indicate whether there is evidence that the effectiveness of the intervention varies according to the proposed modifier.

Towards implementation fidelity, comparisons focusing on differences between the intervention and usual care arms will be made using generalized linear mixed models with logistic link and a random intercept for provider to account for correlation within providers. Comparisons in time, focusing on whether there is a difference between arms in the time a provider spends addressing medication-related issues, will be made using linear mixed models, with a random intercept for provider to account for correlation within providers.

For analysis of program implementation and replication costs, our primary measures of cost will be the average amount of time of clinic staff devoted to the pMTM intervention and usual care and the average cost per-patient for pMTM and usual care. The average time will be calculated for each category of clinic staff, including the pharmacist, by the mean time measured in the audio recordings plus the mean time reported in the annual surveys. The average cost per-patient will convert the average time measures to dollars using Bureau of Labor Statistics data and add in the cost of other clinic resources divided by the number of patients. We will also conduct a sensitivity analysis using different time measures based on the distribution of the time measures across the audio recordings and survey responses.

For all qualitative data, we will employ qualitative content analysis throughout the periods of data collection and analysis [88, 93]. This is appropriate as our goal is to explore the participants’ experiences, focusing on their perceptions of the pMTM intervention and the feasibility and acceptability of the intervention. To achieve this, we will use an inductive coding process in which 2 + research team members independently develop codes and their definitions through reading the transcripts. The team will discuss their respective codes to develop a consolidated codebook. The study team will then independently apply the codebook to the next set of transcripts, and then meet and reconcile their codebooks and coded data. This process will continue until a final codebook is agreed upon. The final codebook will be applied to the remaining transcripts. Coded transcripts will be entered into Atlas.ti version 9.1 for analysis, and we will develop themes that capture the major concepts about feasibility, acceptability, and barriers/facilitators of the pMTM intervention.

### Missing data and intent-to-treat

In the event of missing data, we will examine the data to determine if omission varies by study arm. However, our approach using mixed effects regression modeling will provide accurate estimates and inference in the presence of missing data under certain assumptions. We will check these assumptions and, if necessary, perform sensitivity analyses to quantify the effect of missing outcome data on our results. All outcomes will be analyzed on an intention-to-treat basis.

### Preservation of type-1 error rate

The overall effectiveness of the intervention will be assessed using a multiple degree-of-freedom test with a null hypothesis of no difference between study arms at 90 days post-randomization. Based on our prior studies of pediatric medication regimen complexity, we will adjust for potential confounders including patient age, number of complex chronic conditions, and recent acute healthcare utilization. All quantitative analyses will be performed in Stata 17.0 (College Station, TX). We will use a 2-sided significance level of 0.05 for all hypothesis testing; thus, the type-I error rate for the assessment of overall effectiveness is fixed at 5%. Standard errors and 95% confidence intervals will also be reported.

### Power and sample size

The overall effectiveness of the intervention will be evaluated based on the primary outcome measure, total MRP count. Based on our previous medication safety studies in SCC, we anticipate enrolling 80% of eligible participants and collecting data from ≥ 80% of enrolled participants at the 90-day follow up. We will approach 463 potential participants and enroll 371 to achieve a final analytic sample size of 296 children and their parents. This will provide > 90% power at the 2-sided 0.05 significance level to detect a 1.0 difference between study arms in MRP count, which is sufficient to detect clinically meaningful changes demonstrated by our pilot data. If there is some degree of contamination between the intervention arms due to clinicians seeing patients in both arms, the study will maintain 80% power to detect a significant mean difference in the primary outcome; this assumes a dilution of the treatment effect of 15% (i.e., that the difference in mean outcomes between treatment arms is attenuated to 0.85). These calculations assume a standard deviation in the MRP outcome of 2.6 as determined through prior work in this area. The proposed sample size will also provide adequate power to detect clinically important changes in quantitative secondary outcomes at the 2-sided 0.05 significance level. Assuming a correlation of 0.4 within patients, the study will have 80% power to detect a difference between study arms in mean change of a) PRO-Sx symptom scores by 3.1 points and b) counts of acute healthcare utilization by 0.8–1.1 visits. These calculations assume a correlation of each outcome within patients of 0.4.

To achieve implementation fidelity aims using audiotaped visits, additional sample size and power calculations were performed. With a total of 50 audiotaped visits per study arm, the study will have 80% power to detect a 0.23 difference in proportions if fidelity in the pMTM group is 90%. The study will also have 80% power to detect a mean difference of 2.8 min if the standard deviation of the length of the conversation is 5 min. Correlation within providers will be accounted for by a mixed effects model’s random intercept.

### Data integrity and privacy

This project will produce a variety of data types across the five years of the project. All study data will be collected by trained research personnel during each study phase. Study data will be collected and analyzed from 4 primary sources, including (1) EHR data, (2) prospectively collected patient- and parent-reported data, (3) study visit data, and (4) transcripts from parent and provider interviews. Clinical data will be extracted from the EHR and/or patient charts. Throughout the trial, EHR data will be queried for utilization, pharmacy, and clinical outcome data. We do not anticipate the collection of any paper documents. Raw data will be transformed using REDCap data management tools and the subsequent processed dataset used for statistical analysis. REDCap is a secure, web-based application designed to support data entry, validation and management. Designated research staff will review REDCap data monthly to ensure data completeness and quality. To protect research participant identities and based on ethical and legal considerations, only de-identified individual data will be made available for sharing. All study data will be retained for a minimum preservation time of 3 years. The preservation time will be extended such that resulting publications have been publicly available for at least 12 months before retiring any data. Data will be made available upon request to the larger research community as soon as possible or at the time of associated publication.

### Access to data and dissemination policy

All investigators will have access to the trial’s final dataset. There are no contractual agreements that limit such access. The investigators intend to publish results for all pre-specified primary and secondary outcomes in the peer-reviewed literature, including publication of the study protocol and access to statistical code upon request for review. Dissemination is key to ensuring that any evidence-based practices elucidated from our study can result in substantial improvements in management of pediatric polypharmacy beyond the study’s immediate scope. Study materials, tools, and resources will be developed so that they may be easily adapted to other settings, with particular focus on creation of an implementation and adaptation guide and online training module. Should the pMTM intervention prove effective, we intend to leverage ongoing research partnerships and collaborate with additional sites to test the pMTM intervention on a broader scale.

### Data and safety monitoring board

The study’s principal investigator (JAF) will have overall responsibility for the Data Safety and Monitoring Plan and for participant safety monitoring. As we are studying only the pediatric-implementation of MTM, an evidence-based practice recommended and widely provided for adult and geriatric enrollees with polypharmacy, the risks to human subjects are minimal, Furthermore, any medication-related optimizations made as part of the pMTM intervention are implemented based on joint decision making between the patient, parent, and the PCP during the routine clinical visit. Although minimal risks to human subjects are anticipated and a formal data safety monitoring board is not required, we will take robust precautions to monitor study participants for signal of adverse events or unanticipated problems during the study according to AHRQ requirements.

## Discussion

Optimal health for the priority population of CMC often depends on the chronic use of multiple medications in the outpatient setting. In all populations, MRPs resulting from polypharmacy can lead to potentially devastating outcomes, and CMC are indeed more vulnerable to MRPs. For example, in a study of 144 million pediatric emergency department visits, CMC were approximately 5 times more likely to experience an ADE-related emergency visit [13]. In the outpatient setting, CMC may also have undertreated symptoms, receive suboptimal pharmacotherapy, or experience preventable adverse effects [4, 6, 7, 94]. While pediatric polypharmacy is prevalent, current polypharmacy management strategies are fragmented and reactive, and medication safety initiatives remain a high priority for pediatric complex care programs [33]. The medication-related and overall health outcomes associated with an MTM program for pediatric patients are unknown, particularly as these relate to CMC. We propose that a pMTM intervention by pediatric pharmacists could, through patient-centered medication regimen simplification and tailored caregiver support, address MRPs and result in increased parental confidence and medication understanding, thereby improvement medication safety and effectiveness.

Real and potential limitations of the study do exist. First, enrollment plans were established in alignment with our prior medication safety studies, in which enrollment occurred at a rate of approximately 100 patient-parent pairs per year [4, 6, 7]. If recruitment is slower than planned, we will work with the local family advisory council to alter our recruitment protocol. Also, if the intervention and study instruments are expanded to other languages during the study period, we will include these additional populations. Second, because participant blinding cannot be achieved for the pMTM intervention, participants who do not receive the intervention may leave the study early, potentially biasing results. We will provide small incentives to retain study participants. Also, all study personnel participating in assessment of outcomes, data analysis, and safety monitoring will be blinded. Third, as described above, the risk of contamination is low, but our total sample size accounts for a worst-case scenario of a 15% reduction in treatment effect from contamination. Finally, while we include CMC from a large urban and rural catchment area, this may not be representative of all CMC. To inform generalizability, we will compare enrolled CMC with national data.

The pMTM study is the first randomized controlled trial to evaluate a centralized, coordinated, and comprehensive approach to medication management in CMC with polypharmacy. The results of this trial will quantify the impact of the pMTM intervention on medication safety, effectiveness, and overall medication complexity. Additionally, this trial will examine the impact of pMTM on subsequent acute healthcare utilization by CMC. Through the described systematic approach, the results of this trial will inform the pediatric medical community on the value and effectiveness of pMTM towards optimization of medication therapy among CMC with polypharmacy.

### Trial status

We anticipate that trial recruitment will begin in August 2023 and will be completed by September 2027. The trial protocol is currently active in its original version without revision.

## Supplementary Information

**Additional file 1.** SPIRIT 2013 Checklist: Recommended items to address in a clinical trial protocol and related documents.

## Data Availability

Not applicable.

## Abbreviations

ADE: Adverse drug event
AHRQ: Agency for Healthcare Research and Quality
ARMS: Adherence to Refills and Medications Scale
BSFC: Burden Scale for Family Caregivers
CMC: Children with medical complexity
CMR: Comprehensive medication review
ECG: Electrocardiogram
EHR: Electronic health record
IRB: Institutional review board
MAP: Medication action plan
MRCI: Medication Regimen Complexity Index
MRP: Medication-related problem
MTM: Medication therapy management
NSAID: Non-steroidal anti-inflammatory drug
PATD: Patients’ Attitudes Toward Deprescribing
PCP: Primary care provider
PharmD: Study pharmacist
PHQ-9: Patient Health Questionnaire-9
PML: Personal medication list
PRO-Sx: Parent-Reported Outcomes of Symptoms
SAHL-E: Short Assessment of Health Literacy-English
SCC: Special Care Clinic
